# Identification of a novel *BAAT* frameshift mutation in a female child diagnosed with skeletal dysplasia: A case report

**DOI:** 10.1097/MD.0000000000039509

**Published:** 2024-09-06

**Authors:** Duc Quan Nguyen, Thi Bich Ngoc Can, Chi Dung Vu, Thi Anh Thuong Tran, Ngoc Lan Nguyen, Thi Kim Lien Nguyen, Van Tung Nguyen, Thanh Hien Nguyen, Thi Huong Giang Tran, Huy Hoang Nguyen

**Affiliations:** a Institute of Genome Research, Vietnam Academy of Science and Technology (VAST), Cau Giay, Hanoi, Vietnam; b Department of Endocrinology, Metabolism and Genetic, Center for Rare Diseases and Newborn Screening, Vietnam National Hospital of Pediatrics, Dong Da, Hanoi, Vietnam; c Graduate University of Science and Technology, Vietnam Academy of Science and Technology (VAST), Cau Giay, Hanoi, Vietnam.

**Keywords:** *BAAT* gene, case report, novel mutation, skeletal dysplasia, WES sequencing, X-ray examination

## Abstract

**Rationale:**

Skeletal dysplasias are a complex series of rare genetic disorders that cause irregular development of bones, joints, and cartilages in children. A total of 770 disorders associated with 41 groups of skeletal dysplasia have been documented, demonstrating a wide range of clinical manifestations and varying levels of severity. In addition to conventional methods, whole genome sequencing has emerged as a useful approach to pinpointing the underlying etiology of skeletal dysplasias.

**Patient concerns:**

A 13-month-old female was admitted to the hospital due to the symptoms of jaundice and failure to thrive.

**Diagnoses:**

The child was subjected to blood tests and a radiographic assessment. The blood chemistries revealed elevated levels of total bilirubin (178 µmol/L), bile acids (198 µmol/L), and low levels of serum calcium (1.69 mmol/L) and phosphate (0.8 mmol/L), along with irregular skeletal development in the forearms and legs, considering rickets and cholestasis.

**Interventions:**

Whole exome sequencing data of the proband revealed a homozygous mutation of c.388dupA in the *BAAT* (*bile acid-CoA: amino acid N-acyltransferase*) gene sequence. This mutation caused a frameshift in the amino acid of the BAAT protein, resulting in the pR130Kfs*12 variant. This mutation has been identified as the underlying cause of skeletal dysplasia in the proband.

**Outcomes:**

A novel frameshift mutation in the *BAAT* gene of a Vietnamese female child diagnosed with skeletal dysplasia has been studied by whole exome sequencing analysis.

**Lessons:**

This research reported a case of skeletal dysplasia caused by a frameshift mutation in the *BAAT* gene. The results of this study contribute to our understanding of the diverse factors that influence irregular skeletal development in children and provide genetic data to support clinical practice.

## 1. Introduction

Skeletal dysplasias are a complex group of rare genetic disorders that cause abnormal development of bones, joints, and cartilage in fetuses and children.^[1]^ The prevalence of skeletal dysplasia is about 1/5000 birth incidences, and the severity of this genetic disorder is greatly varied. To date, there have been reports of more than 770 disorders related to skeletal development, which can be categorized into 41 different groups. They exhibit various clinical manifestations and vary in terms of their severity.^[2]^ In mild cases, skeletal dysplasia-affected children appear to have shortened limbs, bowlegs, knock knees, clubfoot, osteoporosis, flat facial features, and/or a prominent forehead. In more severe cases of skeletal dysplasia, the affected children have been diagnosed with serious complications, including thoracic insufficiency syndrome (ribcage problems), spinal stenosis, kyphosis, lordosis, and scoliosis (spine problems), fragile bones, ambiguous genitals, breathing difficulties, and/or hearing impairment.^[2–4]^

Due to their complex phenotypes and individual variations, next generation sequencing has been employed to aid in identifying the causes of skeletal dysplasia at the molecular level. Several genes have been reported as being linked to abnormal skeletal development. For instance, Cissé et al (2024) reported that a specific mutation (c.925C>T; p.Gln309X) on the *RUNX2* (*Runt-related transcription factor 2*) gene, which is responsible for regulating the differentiation of mesenchymal stem cells into bone-forming cells, resulted in severe parietal bone dysplasia in a 20-month-old girl.^[5]^ Nagata et al (2020) identified a novel mutation (c.1133G>A; p.S378N) in the *fibroblast growth factor receptor 3* gene of a 3-year-old girl using whole genome sequencing.^[6]^ The novel mutation has been identified as the cause of achondroplasia in this proband. This genetic change is responsible for the proband’s clinical manifestations of a short-limbed stature, significantly delayed gross motor development, and secondary ossification defects. Hsu et al (2019) identified a pathogenic mutation (p.R594H) in the transient receptor potential vanilloid 4 channel. This mutation has been linked to the development of progressive bowlegs and slightly reduced height in a six-year-old boy.^[7]^ Together, the above data strongly indicate that whole exome sequencing (WES) technology is a cost-effective and advantageous diagnostic method. Therefore, it should be considered a standard procedure for diagnosing skeletal dysplasia, in addition to other conventional methods.

In this study, we report the diagnosis of a Vietnamese girl diagnosed with skeletal dysplasia. WES data revealed that this girl has a novel mutation (c.388dupA) in the sequence of bile acid-CoA: amino acid N-acyltransferase (*BAAT*), leading to the formation of a dysfunctional BAAT (p.S378N) protein. This mutation causes the formation of a premature stop codon, located 12 amino acids downstream of the newly formed amino acid. Our study has once again demonstrated the usefulness of molecular analysis for diagnosing skeletal dysplasias in children.

## 2. Case presentation

### 2.1. Clinical presentation

**Figure 1.**
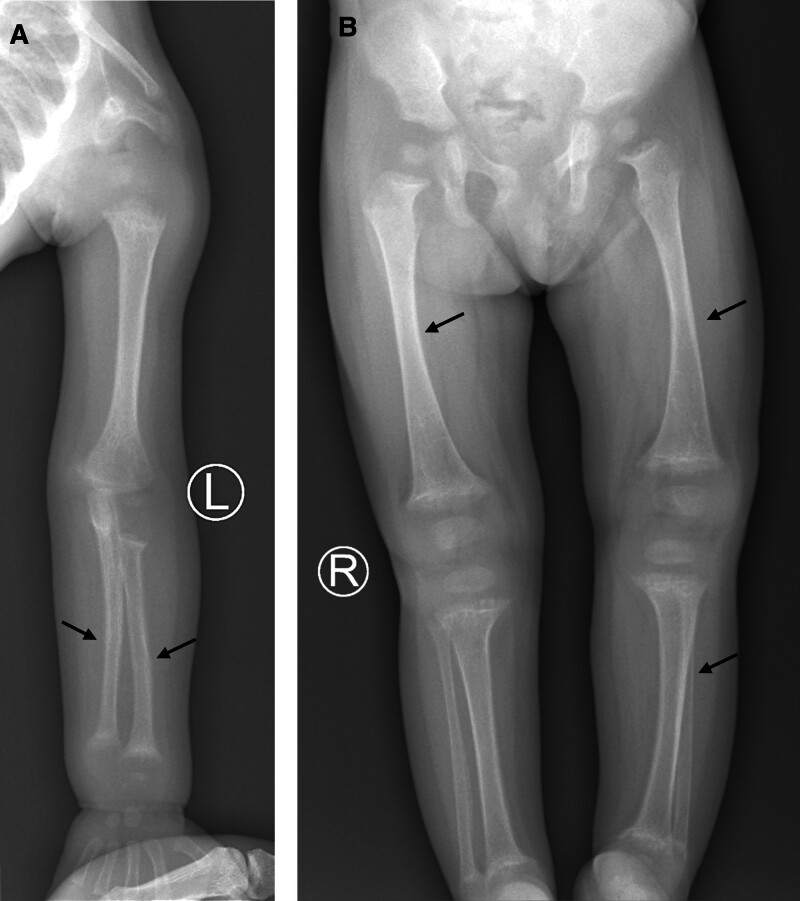
Radiograph assessment of the proband diagnosed with skeletal dysplasia. X-ray images show congenital bowed radius and ulna in the left arm (A), bowed tibia in the left leg, and bowed femur in both legs (B).

The study was approved by the Ethics Committee of the Institute of Genome Research, with the assigned approval number 01-2022/NCHG-HDDD. The proband is a 13-month-old girl who was hospitalized due to presenting symptoms of jaundice and failure to thrive, with a weight of 7.45 kg and a length of 70.5 cm. The blood tests returned increased levels of total bilirubin (178 µmol/L), a pigment that is orange-yellow in color, as well as bile acids (198 µmol/L), and low levels of serum calcium (1.69 mmol/L) and phosphate (0.8 mmol/L). The accumulation of a high level of bilirubin and bile acid indicates that the proband suffered from cholestasis; the condition caused by disorders in bile acid synthesis. Furthermore, skeletal radiographic images, together with low levels of serum calcium (1.69 mmol/L) and phosphate (0.8 mmol/L), demonstrated that the proband suffered from irregular bone development (Fig. 1). Specifically, the radius and ulna in the left arm, the tibia in the left leg, and the femur in both legs of the proband exhibit a bowed shape. The proband received phototherapy to treat the condition of jaundice, and was prescribed vitamin D3 to treat the condition of rickets. However, there is no specific treatment for the skeletal issues. A peripheral blood sample was then taken from the proband for genetic analysis using whole genome sequencing.

### 2.2. Ethics approval and informed consent

This study was conducted in accordance with the Declaration of Helsinki of 2013. The protocol was reviewed and approved by the Ethics Committee of the Institute of Genome Research, approval number 01-2022/NCHG-HDDD. A written consent was obtained from the proband’s parents for conducting genetic analyses and using the obtained data for scientific research and publication.

### 2.3. Molecular investigation

Genomic DNA samples were extracted from peripheral blood samples of the proband using the QIAamp DNA Blood Mini Kit (Qiagen, Germany) according to the manufacturer’s instructions. Qualification and quantification of the genomic DNA sample were determined using gel electrophoresis and a Nanodrop^TM^ spectrophotometer (ThermoFisher Scientific, Waltham, MA, USA). The high-quality DNA sample of the proband was used for whole exome sequencing on the Illumina platform (Illumina, San Diego, CA, USA). The WES data was mapped to the reference human genome GRCh38 by Burrows-Wheeler Aligner (version 0.7.17; RRID: SCR_010910). Variant calling was done by the GATK package (ver. 4.1). A mutation (c.388dupA, p.R130Kfs*12) was identified and predicted to be pathogenic by SIFT (RRID: SCR_012813) and PolyPhen-2 (RRID: SCR_013189) tools. This mutation is not available in any human genome database, including gnomAD v.4.0 (https://gnomad.broadinstitute.org/) and the human gene mutation database (http://www.hgmd.cf.ac.uk/), therefore considering the novel mutation that caused skeletal dysplasia in the proband.

Sanger sequencing was performed for the confirmation of the identified novel mutation in the *BAAT* gene of the proband. The causative mutation of the *BAAT* gene were amplified by polymerase chain reaction using a set of specific primers (F: TGCATATCCGAGCTACAGGC; R: TTACCACATTCTTGTTCAGACCC). The amplified PCR products of the proband and her parents were purified and sequenced on a 3100 Genetic Analyzer (Applied Biosystems, San Francisco, CA) using the BigDye^TM^ Terminator Cycle Sequencing Kit (ThermoFisher, Waltham, MA). The obtained sequencing data were compared with the reference sequence of *BAAT* (NG_009774.1) by the Clustal-Omega online tool (RRID: SCR_001591) using the default parameters.

## 3. Discussion

**Figure 2.**
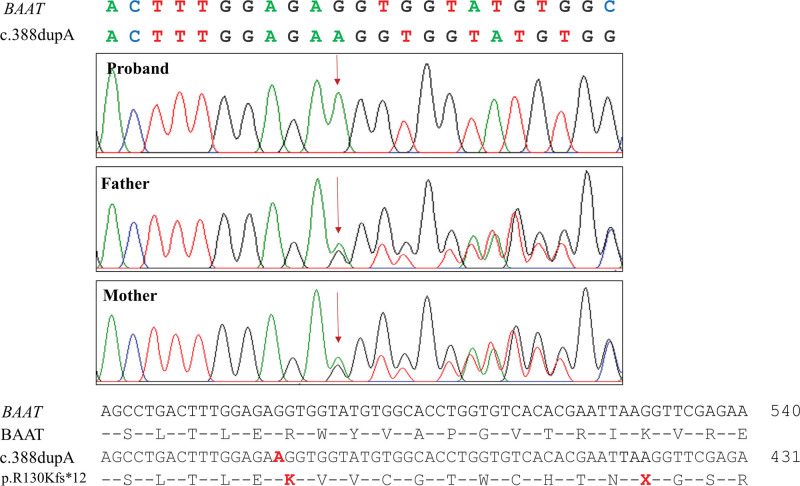
Sanger sequencing results of the proband and the proband’s parents at the mutation site of the *BAAT* gene. The duplication of the adenine nucleotide was found at position 388 (c.388dupA) on the *BAAT* gene sequence, which caused a frameshift at position 130 in the BAAT amino acid sequence (p.R130Kfs*12) and generated a new translation termination at position 12 downstream of the first new amino acid.

In this study, we present a case of a female child who was affected by jaundice, failure to thrive and abnormal limb development. The proband was subjected to whole genome analysis in order to identify the underlying reason for irregular bone formation at the molecular level. WES data revealed a novel homozygous frameshift mutation (c.388dupA, p.R130Kfs*12) in the *BAAT* gene of the proband (Fig. 2).

BAAT is an important liver enzyme involved in the final step of hepatic bile acid conjugation in humans. This process catalyzes the coupling of bile acids with either glycine or taurine, resulting in the formation of T/G-conjugated bile acids.^[8]^ Mutations in the BAAT encoding gene can lead to the disruption of bile acid conjugation. Consequently, individuals suffering from bile acid conjugation deficiency experience symptoms such as hypercholanemia and jaundice. These conditions arise due to the body’s inability to properly absorb dietary fat and lipid-soluble vitamins.^[9]^ *BAAT* mutations have also been associated with the disturbance of bone homeostasis due to the fact that bile acids are capable of regulating the differentiation of osteoblasts and osteocytes as well as the resorption of bone and cartilage matrix through bile acid-activated receptors.^[8]^ A study conducted by Setchell et al reported that a group of 7 patients, ranging from 3 months to 8 years old, suffered from rickets resulting from mutations in the *BAAT* gene.^[9]^ They had been biochemically confirmed to have elevated levels of bile acids, ranging from 248 to 432 μmol/L. Additionally, they were molecularly identified with 4 different types of pathogenic homozygous mutations in the *BAAT* gene, including a nonsense mutation c.58C>T (p.R20X) and 3 missense mutations c1156G>A (p.G386R), c.206A>T (p.D69V), and c.250C>A (p.P84T).^[9]^ In a related study, Carlton et al reported on the case of Amish children who suffered from vitamin deficiency and failure to thrive. This was attributed to a specific mutation in the *BAAT* gene (c.226A>G, p.M76V).^[10]^

The general clinical features of the proband in this study were consistent with those reported in previous studies. The proband’s weight- and length-for-age scores (Z scores) have been determined to be lower than the reference values provided by the World Health Organization.^[11]^ Based on the weight- and length-for-age charts, the proband’s Z scores of weight- and length-for-age were ‐1.7 and ‐1.8, indicating that they fall below the 4.2nd and 3.7th percentiles, respectively. These results indicated that the proband was underweight and short in length. Additionally, the results of the proband’s blood tests revealed the accumulation of bilirubin and bile acid. The bilirubin content was found to be significantly elevated, measuring 2.1 times higher than the normal range of 85.5 µmol/L.^[12,13]^ There has been a significant increase in the bile acid content, reaching levels that are approximately 20.9 times higher than the standard range.^[14]^ In contrast, the levels of serum calcium and phosphate were found to be greatly lower than the normal concentrations. In healthy children, the levels of calcium in ranged from 2.06 to 2.73 mmol/L, while the levels of phosphate ranged from 1.54 to 2.72 mmol/L.^[15,16]^ Furthermore, the guideline set by the American College of Medical Genetics and Genomics has determined that the homozygous frameshift mutation c.388dupA (p.R130Kfs*12) in the *BAAT* gene of the proband is considered a pathogenic mutation, falling under the PSV1, PS2, PM2, and PP3 categories.^[17]^ Sanger sequencing results have confirmed that the proband carries a specific *BAAT* c.388dupA mutation. This mutation was found to be a *de novo* mutation, as it is not inherited from the proband’s parents. Additional functional studies are needed to confirm the pathogenicity of the c.388dupA mutation in the *BAAT* gene.

## 4. Conclusions

In this study, we reported a novel homozygous frameshift mutation (c.388dupA, p.R130Kfs*12) in the *BAAT* gene of a female child diagnosed with skeletal dysplasia. The proband was characterized by developmental delay, along with irregular skeletal development in the left arm and legs. Our findings have provided clinical and genetic information that enriches the understanding of skeletal dysplasia in children.

## Acknowledgments

The authors would like to sincerely thank the Vietnam Academy of Science and Technology for funding our study (Grant number KHCBSS.01/22-24).

## Author contributions

**Conceptualization:** Huy Hoang Nguyen.

**Funding acquisition:** Huy Hoang Nguyen.

**Methodology:** Duc Quan Nguyen, Thi Bich Ngoc Can, Chi Dung Vu, Thi Anh Thuong Tran, Ngoc Lan Nguyen, Thi Kim Lien Nguyen, Van Tung Nguyen, Thanh Hien Nguyen, Thi Huong Giang Tran, Huy Hoang Nguyen.

**Software:** Duc Quan Nguyen, Ngoc Lan Nguyen, Van Tung Nguyen.

**Validation:** Duc Quan Nguyen, Thi Bich Ngoc Can, Chi Dung Vu, Huy Hoang Nguyen.

**Visualization:** Duc Quan Nguyen, Thi Bich Ngoc Can, Ngoc Lan Nguyen, Huy Hoang Nguyen.

**Writing – original draft:** Duc Quan Nguyen, Thi Bich Ngoc Can.

**Writing – review & editing:** Duc Quan Nguyen, Thi Bich Ngoc Can, Chi Dung Vu, Ngoc Lan Nguyen, Thi Kim Lien Nguyen, Van Tung Nguyen, Thanh Hien Nguyen, Thi Huong Giang Tran, Huy Hoang Nguyen.
